# Weyl semimetals in optical lattices: moving and merging of Weyl points, and hidden symmetry at Weyl points

**DOI:** 10.1038/srep33512

**Published:** 2016-09-20

**Authors:** Jing-Min Hou, Wei Chen

**Affiliations:** 1 Department of Physics, Southeast University, Nanjing 211189, China; 2 College of Science, Nanjing University of Aeronautics and Astronautics, Nanjing 210016, China

## Abstract

We propose to realize Weyl semimetals in a cubic optical lattice. We find that there exist three distinct Weyl semimetal phases in the cubic optical lattice for different parameter ranges. One of them has two pairs of Weyl points and the other two have one pair of Weyl points in the Brillouin zone. For a slab geometry with (010) surfaces, the Fermi arcs connecting the projections of Weyl points with opposite topological charges on the surface Brillouin zone is presented. By adjusting the parameters, the Weyl points can move in the Brillouin zone. Interestingly, for two pairs of Weyl points, as one pair of them meet and annihilate, the originial two Fermi arcs coneect into one. As the remaining Weyl points annihilate further, the Fermi arc vanishes and a gap is opened. Furthermore, we find that there always exists a hidden symmetry at Weyl points, regardless of anywhere they located in the Brillouin zone. The hidden symmetry has an antiunitary operator with its square being −1.

In last decade, topological matters have become an important branch of condensed matter physics^1^,^2^. Previously, the studies mainly concentrate on gapped systems, such as integer quantum Hall insualtors^3^ and quantum anomalous Hall insulator^4^, topological insulators^5^, chiral topological superfluids^6^, helical topological superfluids or superconductors^7^, and so on. Recently, physicists pay much attention on the topological characters of gapless systems, which were dubbed as topological semimetals. Generally, topological semimetals include Weyl semimetals^8^,^9^,^10^,^11^,^12^, Dirac semimetals^13^,^14^, topological nodal-line semimetals^10^. For Weyl semimetals, the materials have band structures with band-touching nodal points in momentum space, where the isolated band degeneracy occurs. Near these touching points, the dispersion relation is linear and can be described by a massless two-component Weyl Hamiltonian. At the nodal points, there exist singularities of a Berry field. Integrating the Berry field on the surface enclosing the singular point in momentum space, one obtain a Chern number, i.e., a topological charge. Thus, the band-touching nodal points can be considered as monopoles in momentum space. Due to the Nielsen-Ninomya theorem, the nodal points with opposite topological charges appear in pairs. The meeting of opposite charges in momentum space can lead to annihilation of nodal point pairs. The opposite topological charges can be separated from each other in momentum space so that they cannot be destroyed by the mutual annihilation if the time-reversal symmetry or inversion symmetry is broken. Time reversal symmetry breaking Weyl semimetals were firstly predicted in pyrochlore iridates^8^ and HgCr_2_Se_4_^9^. Recently, inversion symmetry breaking Weyl semimetals were discovered in TaAs family^15^,^16^,^17^,^18^,^19^. In the theoretical aspect, recently, Ganeshan and Das Sarma presented a method to construct a Weyl semimetal by stacking one-dimensional Aubry-Andre-Harper lattice with tight-binding models with nontrivial topology^20^, which provides a theoretical connection between the commensurate Aubry-Andre-Harper model in one dimension and Weyl semimetals in three dimensions.

It is a difficult task to investigate moving and merging of Weyl points and topological phase transitions in real solid materials, the parameters of which can not be tuned in a wide ranges. Fortunately, the high controllability and tunability, and large number of mature detection techniques of cold atoms in optical lattices make them a platform to stimulate many interesting physics in condensed matters. Therefore, it is intriguing to study moving and merging of Weyl points, and topological phase transitions in optical lattices. In recent years, many schemes have been proposed to realize various topological semimetals with neutral atoms in optical lattices. In two dimensions, gapless topological phases were proposed in honeycomb optical lattices^21^,^22^ and square optical lattices^23^,^24^,^25^. The important progress is the realization of topological semimetals in honeycomb optical lattices^26^. In three dimensions, Weyl semimetal were proposed to realized in optical lattices^27^,^28^,^29^,^30^. In order to engineer the topological phases in optical lattices, sometimes, the hopping-accompanying phase, i.e., the Peirls phase, is required. In experiments, the hopping-accompanying phase has been realized with periodic lattice shaking^31^,^32^ and laser-assisted tunneling techniques^33^,^34^,^35^,^36^. Another important progress in experiments is the measurement of Zak phase of topological Bloch bands in optical lattices^37^, which provides a path to detect topological characters in optical lattices.

In this paper, we design a cubic optical lattice trapping cold fermionic atoms, which can be realized based the laser-tunnelling technique^33^,^34^,^35^,^36^. In different parameter ranges, the system supports three classes of Weyl semimetals, one of which has two pairs of Weyl points in the Brillouin zone, the other two have one pair of Weyl points in the Brillouin zone. By adjusting the parameters, we can study the moving and merging of Weyl points. When Weyl points with opposite topological charges meet together, they annihilate and a topological phase transition happens. We also investigate the Fermi arc of surface states of a (010) slab. Fermi arcs connect the projections of Weyl poionts on the surface Brillouin zone and evolve with the moving of Weyl points. For the Weyl semimetal phase with two pairs of Weyl points, there are two Fermi arcs connect projections of Weyl points with opposite charges on the surface Brillouin zone. When a pair of Weyl points annihilate, the two Fermi arcs link into one single Fermi arc connecting the projections of the remaining Weyl points. We find that the band degeneracy at Weyl points implies a hidden symmetry that has an antiunitary operator with its square being −1. Based on a mapping method, we discover the hidden symmetry at each Weyl point in the Brillouin zone and discuss its relation with topological phase transitions.

## Results

### Weyl semimetals in optical lattices

**Figure 1:**
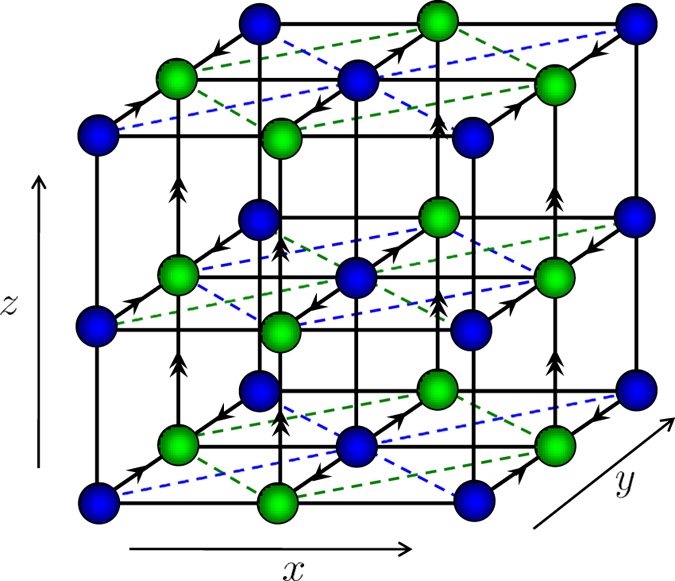
Schematic of the cubic optical lattice. Here, the blue and green balls represent sublattices A and B, respectively; the single arrows and double arrows denote *π*/2 and *π* phases along with the hopping, respectively.

Here, we consider a cubic optical lattice as shown in Fig. 1, where the arrows represent the hopping-accompanying phase. The hopping-accompanying phase is *π*/2 for the hopping along the *y* axis and *π* for the *z* axis. Due to the appearing of the hopping-accompanying phases, the translation symmetry is broken. Thus the lattice is divided into two sublattices, i.e. sublattices *A* and *B*. Assuming the distance between the nearest lattice sites being 1, we define the primitive lattice vectors as a_1_ = (1, −1, 0), a_2_ = (1, 1, 0), and a_3_ = (0, 0, 1). The primitive reciprocal lattice vectors are b_1_ = (*π*, −*π*, 0), b_2_ = (*π*, *π*, 0), and b_3_ = (0, 0, 2*π*). Besides the hopping between nearest lattice sites, we also consider the diagonal hopping in the *x*−*y* plane and a staggered potential. The corresponding Hamiltonian is *H* = *H*_0_ + *H*_*d*_ + *H*_*s*_ with


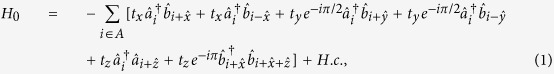


and


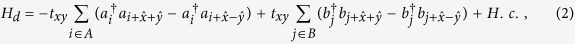


and


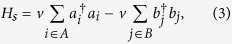


where *a*_*i*_ and *b*_*i*_ are the annihilation operators destructing a particle at a lattice site of sublattice *A* and *B*, respectively; *t*_*x*_ and *t*_*y*_ represent the amplitudes of hopping along the *x* and *y* directions, respectively; *t*_*xy*_ denotes the amplitude of hopping along the diagonal direction; *v* represents the magnitude of the staggered on-site potential. This optical lattice can be realized through the laser-assisted tunneling technique, which has been applied in several experiments^33^,^34^,^35^,^36^.

**Figure 2:**
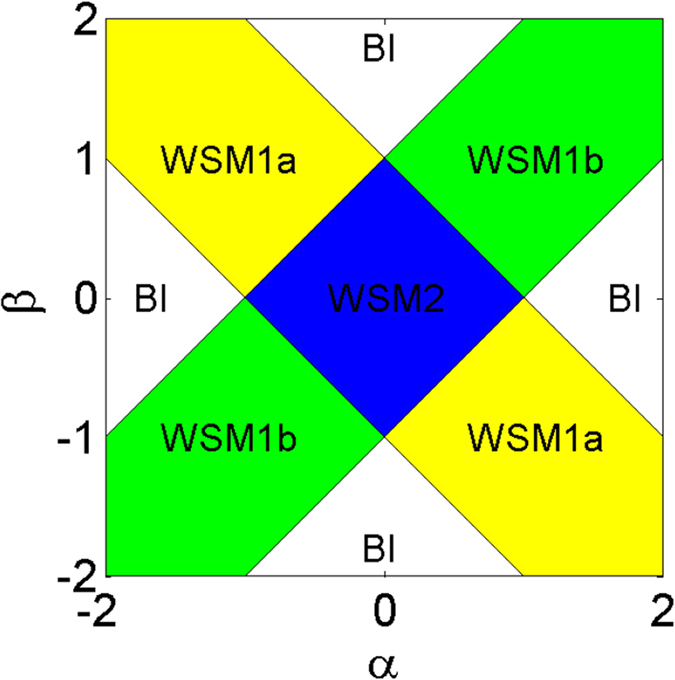
Schematic of the phase diagram. Here, WSM2 (blue) denotes the Weyl semimetal phase with two pairs of Weyl points in the Brillouin zone; WSW1a (yellow) and WSW1b (green) denote the two semimetal phases with a pair of Weyl points in the Brillouin zone; BI denotes the band insulator phase.

Taking the Fourier’s transformation on Equations (1), (2) and (3), we rewritten the Hamiltonian as 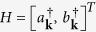

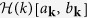
, where 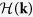 is the corresponding Bloch Hamiltonian as


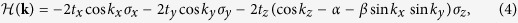


with *α* = *v*/2*t*_*z*_ and *β* = 2*t*_*xy*_/*t*_*z*_ being the dimensionless parameters and *σ*_*x*_, *σ*_*y*_ and *σ*_*z*_ being the Pauli matrices defined in the sublattice space. Diagonalizing Equation (4), we obtain the corresponding dispersion relation as


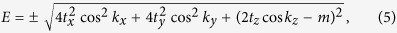


with *m* = 2*t*_*z*_(*α* + *β* sin *k*_*x*_ sin *k*_*y*_). From this dispersion relation, we can see that two bands touch at some points **W**_*i*_ in the Brillouin zone in some parameter ranges. Near the touching points, the dispersion relation has the linear form as


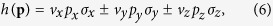


with **p** = **k** − **W**_*i*_. Around the the touching points, the chirality can be defined as


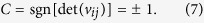


which is also the topological charge at Weyl points. Thus, the touching points are Weyl points and, correspondingly, the system is a Weyl semimetal phase. According to the number of Weyl points in different parameter ranges, we can classify the system into four phases: (i) When 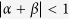 and 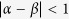 are satisfied, there are four distinct points 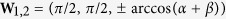 and 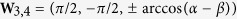 in the Brillouin zone. Since there are two pairs of Weyl points in the Brillouin zone, we term this phase as WSM2 phase. (ii) For the case 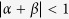 and 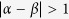, only the pair 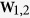 exists. Thus, we term this phase as WSM1a phase. (iii) For the case 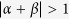 and 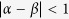, where the Weyl points **W**_3,4_ still remain. We term this new phase as WSM1b phase, which is different from the WSM1a phase. (iv) For 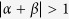 and 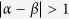, no Weyl point exists and a gap opens, so the system is a band insulator. The phase diagram is shown as in Fig. 2.

### Moving and merging of Weyl points, topological phase transition, and Fermi arcs of surface states

Here, we investigate moving and merging of Weyl points along with varying of the dimensionless parameters *α* and *β*. Merging of Weyl points and annihilations of topological charges lead to topological phase transitions. In our model, there are four kinds of topological phase transitions such as (i) transition from the WSM2 phase to the MSM1a phase, (ii) transition from the MSM2 phase to the MSM1b phase, (iii) transition from the MSM1a phase to the band insulator phase, and (iv) transition from the MSM1b phase to the band insulator phase.

**Figure 3:**
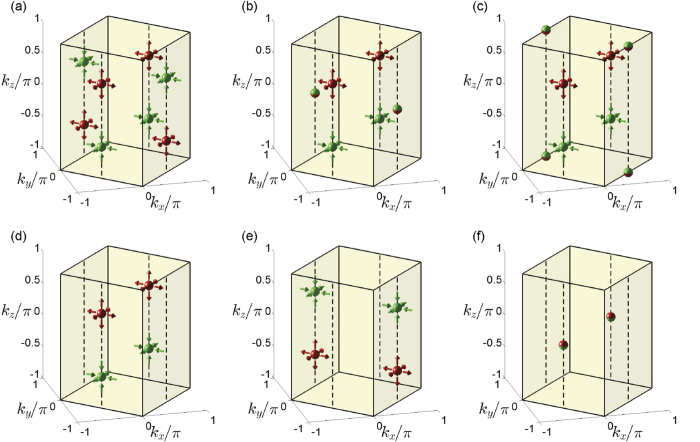
Weyl points in Weyl semimetal phases for. (**a**) The WSM2 phase with *α* = 0 and *β* = 0, (**b**) the boundary between the MSM2 phase and the MSM1a phase with *α* = 0.5 and *β* = −0.5, (**c**) the boundary between the MSM2 phase and the MSM1a phase with *α* = −0.5 and *β* = 0.5, (**d**) the MSM1a phase with *α* = 0.8 and *β* = −0.8, (**e**) the MSM1b phase *α* = 0.8 and *β* = 0.8, (**f**) the boundary between the MSM1a phase and the band insulator phase with *α* = 1.3 and *β* = −0.3. For all of cases, we have set *t*_*x*_ = *t*_*y*_ = *t*_*z*_ = *t*. The yellow bulks represent the Brillouin zone; the red and green balls represent the Weyl points with positive and negative topological charges (also denoted by all-out and all-in arrows), respectively; the half red and half green balls represent merged Weyl points.

The WMS2 phase has two pairs of Weyl points **W**_1,2_ and **W**_3,4_ with topological charges *C*_1,2_ = ±1 and *C*_3,4_ = ±1, as shown in Fig. 3(a). When we keep *α* + *β* invariant and increase *α* − *β*, the Weyl points **W**_3,4_ move towards each other and **W**_1,2_ stay at the original positions. When *α* − *β* increases to 1, the Weyl points meet at (*π*/2, −*π*/2, 0) in the Brillouin zone and merge, as shown in Fig. 3(b). When *α* − *β* further increases more than 1, the Weyl points **W**_3,4_ annihilate and only **W**_1,2_ remain, the system from the MSM2 phase turns into the MSM1a phase, i.e., topological phase transition (i) happens. Topological phase transition (i) can also occur through the other type of moving and merging of Weyl points. Starting from the WSM2 phase, we keep *α* + *β* invariant and decrease *α* − *β*, the Weyl points **W**_3,4_ move away from each other and **W**_1,2_ stay at their starting positions. When *α*−*β* decreases to −1, the Weyl points **W**_3,4_ arrive at (*π*/2, −*π*/2, ±*π*), which are identical points in the Brillouin zone, i.e., **W**_3,4_ meet and merge, as shown in Fig. 3(c). When *α*−*β* is less than −1, **W**_3,4_ annihilate and topological phase transition (i) happens. When it arrives at the MSM1a phase, there exist only one pair of Weyl points **W**_1,2_, which have opposite topological charges, in the Brillouin zone, as shown in Fig. 3(d). Similarly, there are two types of moving and merging of Weyl points to realize topological phase transition (ii), i.e., the transition from the WSM2 phase to the WSM1b phase. We can vary the value of *α* + *β* and keep *α*−*β* invariant. When *α* + *β* increases to 1 or −1, the Weyl points **W**_1,2_ meet and merge at the center or the surface of the Brillouin zone, and **W**_3,4_ still remain. When |*α* + *β*| is greater than 1, the Weyl points **W**_1,2_ annihilate and a topological phase transition from the MSM2 phase into MSM1b phase happens, as shown in Fig. 3(e). For the MSM1a phase, we can also increase |*α* + *β*| to 1, the remaining Weyl points **W**_1,2_ meet and merge at the center or the surface of the Brillouin zone, as shown in Fig. 3(f). If we further increase |*α* + *β*| greater than 1, the remaining Weyl points **W**_1,2_ annihilate and a gap opens, topological phase transition (iii) happens. For the MSM1b phase, if we increase |*α* − *β*| to 1, the remaining Weyl points **W**_3,4_ meet and merge at the surface or corner of the Brillouin zone. If we further increase |*α* − *β*| greater than 1, the remaining Weyl points **W**_3,4_ annihilate and a gap opens, so topological phase transition (iv) happens. In all the topological phase transitions, it is found that topological charges respect a conservation law and they are only created and annihilated in pairs.

**Figure 4:**
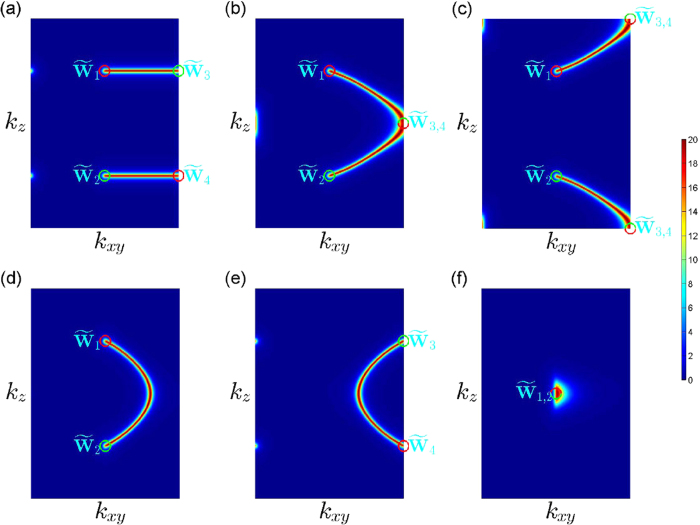
The spectral functions of surface states with E = 0 on the (010) surface Brillouin zone for. (**a**) The WSM2 phase with *α* = 0 and *β* = 0, (**b**) the transition from the MSM2 phase to the MSM1a phase with *α* = 0.5 and *β* = −0.5, (**c**) the transition from the MSM2 phase to the MSM1a phase with *α* = −0.5 and *β* = 0.5, (**d**) the MSM1a phase with *α* = 0.8 and *β* = −0.8, (**e**) the MSM1b phase *α* = 0.8 and *β* = 0.8, (**f**) the transition from the MSM1a phase to the band insulator phase with *α* = 1.3 and *β* = −0.3. For all of cases, we have set *t*_*x*_ = *t*_*y*_ = *t*_*z*_ = *t*. Here, the rectangles represent the (010) surface Brillouin zone and *k*_*xy*_ is the component on the *xy* plane in momentum space for the wavevectors on the surface Brillouin zone. The red and green circles represent the projections of Weyl points with positive and negative topological charges on the surface Brillouin zone; the half red and half green circles represent the projections of merged Weyl points on the surface Brillouin zone.

In order to further study the characters of Weyl semimetals and topological phase transitions, we calculate the surface states of a slab geometry with (010) surfaces and investigate the evolution of Fermi arcs along with the moving and merging of Weyl points. In Fig. 4, we show the spectral function of the surface states at zero energy. The spectral function can be calculated through the formula 
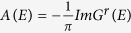
, where *G*^*r*^(*E*) is the retarded Green function of the system. The projections of Weyl points **W**_1,2,3,4_ on the surface Brillouin zone are denoted as 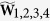. Figure 4(a) shows that, in the WSM2 phase, there are two Fermi arcs in the surface Brilllouin zone, which connect points 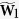 and 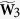, 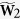 and 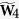, respectively. Since Weyl points **W**_1_ and **W**_3_, **W**_2_ and **W**_4_ have opposite topological charges, we conclude that Fermi arcs connect the projections of Weyl points with opposite topological charges on the surface Brillouin zone. When |*α*−*β*| increases to 1, 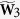 and 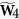 meet and merge at the side boundary or the corners of the surface Brillouin zone, thereby two Fermi arcs combine into one single Fermi arc, as shown in Fig. 4(b,c), which corresponds to topological phase transition (i). When |*α*−*β*| increases greater than 1, the system in the MSM1a phase, the Fermi arc connects the projections of the remaining Weyl points 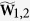, as shown in Fig. 4(d). Similarly, for the MSM1b phase, there exists a Fermi arc connect the points 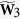 and 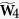 in the surface Brillouin zone, as shown in Fig. 4(e). When the transition from the MSM1a phase or the MSM1b phase to the band insulator phase the happen, the Fermi arc firstly shrink into a point, as shown in Fig. 4(f), and finally disappears.

### Hidden symmetry at Weyl points

Here, we build the hidden symmetry at Weyl points. For convenience to construct the hidden symmetry, we suppose the case with the Hamiltonian *H*_0_ as Eq. (1) as the original model and the total model *H* = *H*_0_ + *H*_*d*_ + *H*_*s*_ as the modified model.

#### Hidden symmetry at Weyl points of the original model

In the following, we will show that the Weyl points in the original model are protected by a hidden symmetry. For the original model, the lattice is invariant under the operation defined as


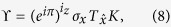


where *K* is the complex conjugate operator; 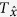 is a translation operator that moves the lattice along the *x* direction by a unit vector; *σx* is the Pauli matrix representing the sublattice exchange; 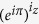 is a local *U*(1) gauge transformation. It is easy to prove that the symmetry operator 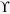 is antiunitary, and its square is equal to 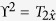.

By setting *α* = 0 and *β* = 0, the Bloch Hamiltonian of original model can be obtain from Eq. (4) as


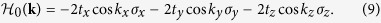


The symmetry operator 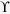 can be considered as a self-mapping of the original model defined as


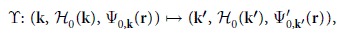


where 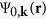 and 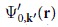 are the Bloch functions of the original model. We suppose that the Bloch functions of the square lattice model have the form as


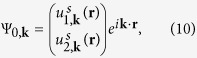


with 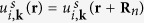 with *i* = 1, 2 for two sublattices and **R**_*n*_ being a lattice vector. Performing the symmetry transformation on the Bloch function (10) leads to 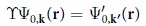 with 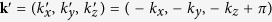. If the condition 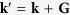, where **G** is the reciprocal lattice vector, is satisfied, **k** is a 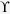-invariant point. In the Brillouin zone, the distinct 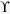-invariant points are 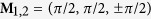, 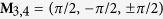, 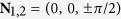, and 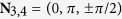. The square of the 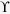 operator can be written in the form as 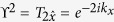 in the Bloch representation. It is easy to verify that 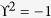 the points **M**_*i*_(*i* = 1, 2, 3, 4), while 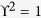 at the points **N**_*i*_(*i* = 1, 2, 3, 4). Considering the antiunitarity of the operator 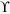, based on Kramers theorem, we can conclude that there must be band degeneracies at the 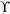-invariant points **M**_*i*_(*i* = 1, 2, 3, 4), which are just the Weyl points **W**_*i*_(*i* = 1, 2, 3, 4) in the MSM2 phase with *α* = 0 and *β* = 0. There, a hidden symmetry with its square of operators being −1 exists at the Weyl points of the original model.

#### Hidden symmetry at Weyl points of the modified model

It is easy to verify that the hidden symmetry 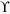 is violated in the modified model. However, with the mapping Ω*_α_*,_*β*_ from the modified model into the original model defined in section Methods, we can find the hidden symmetry in the modified model. Based on the mapping Ω_*α*,*β*_, we define an operation 
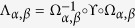
, which can be regarded as a self-mapping of the modified model as


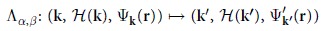


Performing the above operation on the Bloch function of the modified model, we have 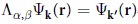, where 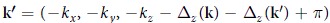 with 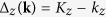 being the shift of the *z*-component of the wave vector **k** due to the mapping Ω_*α*,*β*_. If 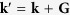 is satisfied, **k** is a 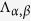-invariant point. In the Brillouin zone, the distinct 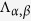-invariant points are 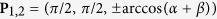, 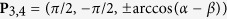, 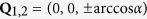 and 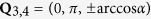.

From the definition of the operator 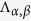, we can verify 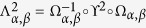, which acts on the Bloch function as 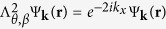. Substituting the 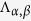-invariant points 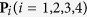 and 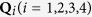 into the above equation, we find 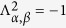 at 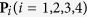, while 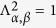 at 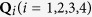. Since 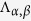 is an antiunitary operator, based on Kramers theorem, there must exist band degeneracies at the 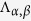-invariant points 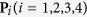, which are just the Weyl points 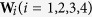 of the WSM2 phase for 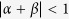 and 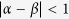. For 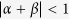 and 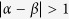, **P**_3,4_ do not exist, there are only the 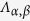-invariant points **P**_1,2_, which correspond to the Weyl points **W**_1,2_ of the WSM1a phase. Similarly, for 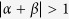 and 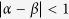, **P**_1,2_ do not exist, there are only the 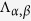-invariant points **P**_3,4_, which correspond to the Weyl points **W**_3,4_ of the WSM1b phase. For the case 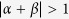 and 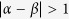, all the 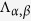-invariant points **P**_1,2,3,4_ do not exist, so there are not Weyl points, which corresponds to a band insulator phase.

**Figure 5:**
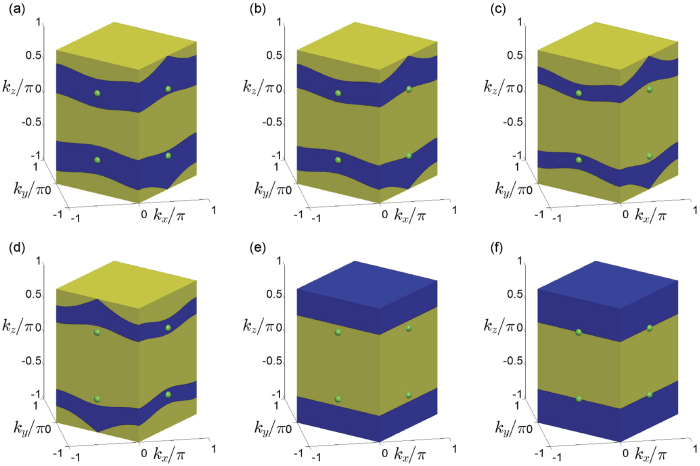
The mapping from the Brillouin zone of the modified model into the Brillouin zone of the original model for. (**a**) *α* = −0.3, *β* = 0.3; (**b**) *α* = 1, *β* = −0.8; (**c**) *α* = 1, *β* = 0.8; (**d**) *α* = 2, *β* = 0; (**e**) *α* = 0.6, *β* = −0.4; (**f**) *α* = 1, *β* = 0;. Here, the yellow bulk represents the Brillouin zone of the original model; the green balls mark the Weyl points of the original model; the blue part represent the image of the mapping in the Brillouin zone of the original model for the Brillouin zone of the modified model.

We can interpret the above results in an intuitive way. The mapping Ω_*α*,*β*_ from the Brillouin zone of the modified model into that of the original model is not surjective, which can be seen in Fig. 5. For the WSM2 phase, i.e. 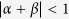 and 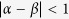, the image of the mapping for the Brillouin zone of the modified model covers the degenerate 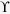-invariant points **M**_1,2_ and **M**_3,4_ in the Brillouin zone of the original model, as shown in Fig. 5(a). Therefore, there are always two pairs of 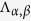-invariant points **P**_1,2_ and **P**_3,4_, where the Weyl points locate, map into the degenerate 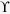-invariant points **M**_1,2_ and **M**_3,4_. When we increase 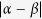 to 1, **P**_3_ and **P**_4_ become the same point of the Brillouin zone of the modified model, which maps into the points **M**_3,4_ in the Brillouin zone of the original model as shown in Fig. 5(b), so the corresponding two Weyl points merge and a phase transition from the WSM2 phase to the WSM1a phase occurs. When 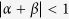 and 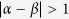, the image of mapping for the Brillouin zone of the modified model only covers the degenerate 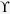-invariant points **M**_1,2_ in the Brillouin zone of the original model as shown in Fig. 5(c). There exists a pair of Weyl points **P**_1,2_ in the Brillouin zone of the modified model mapping into the degenerate 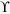-invariant points **M**_1,2_, which corresponds to the WSM1a phase. Similarly, when 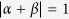 and 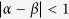, the image of the mapping for the Brillouin zone of the modified model covers **M**_1,2_ and **M**_3,4_ while **M**_1,2_ locate at the edge of the image and the same point in the Brillouin zone of the modified model maps into **M**_1,2_. The degenerate 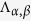-invariant points **P**_1,2_ merge at the edge of the Brillouin zone of the modified model while **P**_3,4_ still exist. This case corresponds to the phase boundary between the WSM2 and WSM1b phases. When 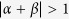 and 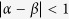, the image of the mapping for the Brillouin zone of the modified model only covers the degenerate 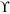-invariant points **M**_3,4_ as shown in Fig. 5(d). Correspondingly, there exists a pair of Weyl points **P**_3,4_ in the Brillouin zone of the modified model mapping into the degenerate 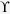-invariant points **M**_3,4_, which corresponds to the WSM1b phase. When 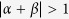 and 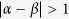, the image of mapping for the Brillouin zone of the modified model does not cover any degenerate 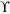-invariant points in the Brillouin zone of the original model as shown in Fig. 5(e). Therefore, there does not exist any Weyl point in the Brillouin zone of the modified model and a gap opens, which corresponds to the band insulator phase. When 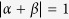 and 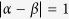, a direct phase transition between the MSM2 phase and the band insulator phase occurs, where two pairs of Weyl points merge simultaneously. For this case, the edge of the image of the mapping for the Brillouin zone of the modified model covers the degenerate 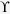-invariant points **M**_1,2_ and **M**_3,4_ in the Brillouin zone of the original model as shown in Fig. 5(f), which means that the four 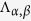-invariant **P**_1,2_ and **P**_3,4_ merge as two points. Therefore, the two pairs of Weyl points simultaneously merge at the edge of the Brillouin zone of the modified model.

## Discussion

In summary, we have proposed a scheme to realize Weyl semimetals in a cubic optical lattice. There exist three Weyl semimetal phases, such as the WSM2, WSM1a, and WSM1b phases, for different parameter ranges. In the Brillouin zone, there are two pairs of Weyl points for the WSM2 phase while there is one pair of Weyl points for the MSM1a and MSM1b phases. The Weyl points move along with varying of the parameters. When the Weyl points with opposite topological charges meet, they merge and annihilate, which leads to a topological phase transition. The spectral functions of surface states at zero energy for a slab with (010) surfaces have been calculated. Fermi arcs appear to connect the projection of the Weyl points with opposite topological charges on the surface Brillouin zone. There are two Fermi arcs in the WSM2 phase and there is one in the MSM1a and MSM1b phases. When the phase transition from the WSM2 phase to the MSM1a or MSM1b phase happens, the two Fermi arcs combine into one Fermi arc. For the phase transition from the MSM1a or MSM1b phase to the band insulator phase, the Fermi arc shrinks into a point, then disappears. We also found that there exist hidden symmetries at all of Weyl points. These hidden symmetries have an antiunitary operator with its square being −1. Based on the mapping method^25^, we constructed hidden symmetries at all of Weyl points. Our work deepens our understanding of Weyl semimetals on the point view of symmetry.

## Methods

### The mapping from the modified model into the original model

We can define a mapping from the modified model into the original model as^25^


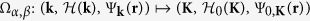


where 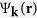 represents the Bloch function of the modified model. The concrete form of the mapping 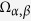 depends on the dimensionless parameters with 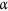 and 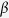. For this mapping, we have 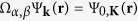 with


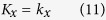



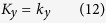



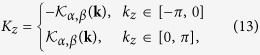


where 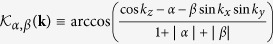. Replacing **k** in equation (4) with **K** via Equations (11), (12), and (13), we obtain


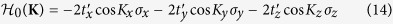


with 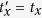, 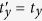, and 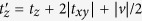, which is just the Bloch Hamiltonian (9) of the original model.

## Additional Information

**How to cite this article**: Hou, J.-M. and Chen, W. Weyl semimetals in optical lattices: moving and merging of Weyl points, and hidden symmetry at Weyl points. *Sci. Rep.* **6**, 33512; doi: 10.1038/srep33512 (2016).
